# Progression of Lumbar Spine Degeneration After Laminectomy

**DOI:** 10.7759/cureus.76097

**Published:** 2024-12-20

**Authors:** Kunihiko Hashimoto, Kazuma Kitaguchi, Daisuke Tateiwa, Kazuya Oshima, Eiji Wada

**Affiliations:** 1 Department of Orthopedic Surgery, Spine and Spinal Cord Center, Osaka International Medical and Science Center, Osaka, JPN; 2 Department of Orthopedic Surgery, Sumitomo Hospital, Osaka, JPN

**Keywords:** degenerative lumbar spinal disease (dlsd), laminectomy, lumbar canal stenosis (lcs), reoperation, surgical procedures

## Abstract

Introduction: Lumbar canal stenosis (LCS) is a common degenerative lumbar spinal disease (DLSD) widely treated by decompression surgery, also known as laminectomy. Few cases have been observed where DLSD has progressed postoperatively, thus requiring reoperation. However, data on such cases are limited.

Methods: We included 247 patients (148 men and 99 women; mean age = 73.3 years) with a mean follow-up of 2.3 years in this single-center retrospective study. Among them, 129 patients underwent bilateral partial laminectomy (BPL), 91 patients underwent lumbar spinous process-splitting laminectomy (LSPSL), and 27 underwent microendoscopic laminotomy (MEL).

Results: Of all the patients, 34 (13.8%) exhibited progression of lumbar spine degeneration symptoms, with nine (3.6%) requiring reoperation. Over 90% of new symptoms developed within one year of the initial surgery. Reoperation rates were significantly higher in patients with foraminal stenosis (P = <0.001). Additionally, 35 patients (14.2%) exhibited slippage progression. LSPSL resulted in significantly less slippage progression (P = 0.026). Spinal canal and foraminal stenosis were significantly associated with slippage progression (P< 0.001 and P = 0.010, respectively).

Conclusions: LSPSL reduced the incidence of canal and foraminal stenosis. Symptomatic DLSD was more common within one year post surgery, with foraminal stenosis more frequently requiring reoperation.

## Introduction

Lumbar canal stenosis (LCS) is a common degenerative lumbar spinal disease (DLSD) treated by decompression surgery, also known as laminectomy. Although the symptoms (back and lower limb pain, motor weakness, numbness, and intermittent claudication) of most patients with LCS are improved after lumbar posterior decompression, a subset may experience recurrent symptoms due to DLSD progression. One known cause of DLSD progression is postoperative spinal instability [1], often resulting from intraoperative injury to the paravertebral muscles or posterior elements (including the laminae, spinous processes, interspinous ligaments, and facet joints) during conventional laminectomy [2-4]. Thus, various techniques, such as bilateral partial laminectomy (BPL) [5-7], lumbar spinous process-splitting laminectomy (LSPSL) [8-12], microscopic bilateral spinal decompression via unilateral laminotomy [13], and microendoscopic laminotomy (MEL) [14-17], have been developed to make the lumbar posterior decompression procedure less invasive. However, few studies have reported on postoperative degeneration and the need for reoperation in such cases [6,15].

In this study, we focused specifically on the progression of lumbar spine degeneration, reoperation after laminectomy, and the related factors.

## Materials and methods

Patients

**Figure 1:**
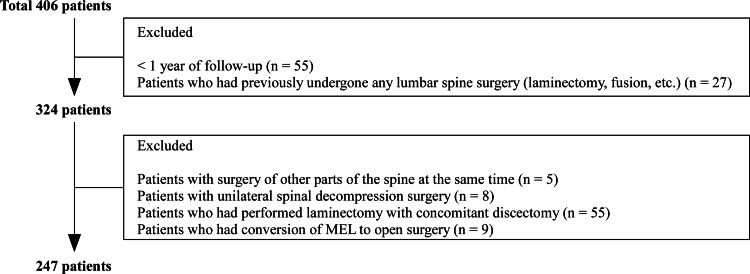
Flowchart showing patient exclusion criteria. MEL: microendoscopic laminotomy.

Between January 2018 and December 2022, we retrospectively reviewed the medical records of 247 patients (148 men and 99 women; mean age = 73.3 ± 9.3 years; range = 38-93 years) at a single institution. These patients underwent either BPL (129 patients), LSPSL (91 patients), or MEL (27 patients) without concomitant discectomy for LCS, including lumbar spondylolisthesis. All patients had at least one year of follow-up (mean duration = 2.3 ± 1.1 years; range = 1-6 years; Table 1). We obtained informed consent from all patients, and the institutional review board approved this study. The 159 patients excluded in this study are shown in Figure 1.

**Table 1:** Characteristics of the patients. BPL, bilateral partial laminectomy; LSPSL, lumbar spinous process-splitting laminectomy; MEL, microendoscopic laminotomy.

Characteristic	Value
Total number of cases	247
Mean age at operation (range), years	73.3 ± 9.3 (38–93)
Sex (male: female)	148:99
Duration of follow-up (range), years	2.3 ± 1.1 (1.0–6.0)
No. of decompression levels	
1	111
2	97
3, 4, 5	39
Surgical methods	
BPL	129
LSPSL	91
MEL	27
Mean operation time (range), minutes	136.1 ± 55.5 (50–381)
Mean blood loss (range), g	82.4 ± 98.5 (0–530)
Progression of lumbar spine degeneration	34
Reoperation	9

DLSD and instability

Progression of lumbar spine degeneration was defined as the development of disc herniation, spinal canal stenosis, foraminal stenosis, or facet joint cysts. It was characterized by acute lower limb pain and numbness, with or without intermittent claudication that developed postoperatively and accompanied by neurological symptoms matching magnetic resonance imaging findings (at the same spinal level as the initial surgery).

Postoperative spinal instability was defined as slippage progression, indicated by a change of 3 mm or more between the operation and symptom onset (or the last consultation day) on standing radiographs [18,19]. It was evaluated separately from the progression of degenerative lumbar spine symptoms.

Surgical procedures

**Figure 2:**
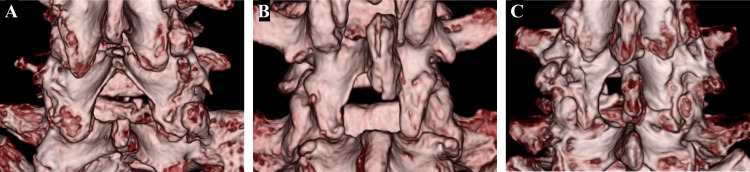
Three-dimensional computed tomography images after surgery. Three-dimensional computed tomography images after bilateral partial laminectomy (A), lumbar spinous process-splitting laminectomy (B), and microendoscopic laminotomy (C).

The procedure of BPL was reported by Eule et al. [5]. The paravertebral muscles were detached from the spinous processes and lamina to the medial facet joints. The distal lamina and spinous processes of the upper vertebra and the proximal lamina and spinous processes of the lower vertebra were removed using chisels and Kerrison rongeurs at the decompression level. The ligamentum flavum was excised in a proximal-to-distal direction and laterally, while the facet joints remained intact (Figure 2A).

LSPSL is a lumbar posterior decompression procedure that reduces the risk of iatrogenic soft tissue injury, as reported by Watanabe et al. [8]. The proximal spinous process was split longitudinally at its midline. The spinous process was then separated from the proximal lamina at its base, while the bilateral paravertebral muscles remained attached to the lateral aspect of the split spinous process. Muscles attached to the proximal and distal laminae were detached. Partial laminectomy and ligamentum flavum resection were performed to decompress the spinal canal. The split spinous process was reconstructed using resorbable sutures (Figure 2B).

MEL has been developed to preserve the paravertebral muscles and the posterior elements. It was performed using the paramedian general approach [15]. A 1-2 cm long incision was made lateral to the spinous process, serial tubal dilators were subsequently inserted, and a tubular retractor was placed. The endoscope was attached to the tubular retractor and any residual muscle and soft tissues overlying the lamina and facet joints were removed. Laminotomy was performed from the base of the spinous process to secure the contralateral surgical field. Decompression of the bilateral lateral recesses was achieved by medial trumpet facetectomy using a high-speed drill and Kerrison rongeurs. The ligamentum flavum was detached from the laminae and removed (Figure 2C).

Statistical analyses

All statistical analyses, unless otherwise indicated, were performed using the Excel statistical software package Bell Curve for Excel (Social Survey Research Information Co., Ltd., Tokyo, Japan). All results are presented as means ± standard deviation. Intergroup comparisons were analyzed using the Mann-Whitney and Fisher’s exact tests. Contingency tables were analyzed using the chi-square test and residual analysis. Statistical significance was set at P < 0.05.

## Results

Characteristics of the patients who progressed lumbar spine degeneration

A total of 34 patients (13.8%) exhibited postoperative progression of DLSD at the same spinal level, while the remaining 213 patients did not exhibit progression. No significant differences existed between both groups in terms of age at the time of surgery, follow-up duration, sex ratio, number of decompression levels, surgical procedures, operative time, or blood loss volume (Table 2). Out of the study population, nine patients (3.6%) underwent reoperation at the same postoperative spinal level. No significant differences were observed between both reoperation and non-reoperation groups in terms of age at the time of surgery, follow-up duration, sex ratio, number of decompression levels, surgical methods, operative time, or blood loss volume (Table 3).

**Table 2:** Characteristics of the patients who progressed lumbar spine degeneration. Op, operation; BPL, bilateral partial laminectomy; LSPSL, lumbar spinous process-splitting laminectomy; MEL, microendoscopic laminotomy. Mean values are presented as mean ± SD.

Characteristic	Progression of lumbar spine degeneration	Non-progression of lumbar spine degeneration	P-value
Mean age at op (range), years	73.1 ± 9.2 (50–88)	73.3 ± 9.3 (38–93)	0.977
Sex (male: female)	19:15	129:84	0.707
Duration of follow-up (range), years	2.4 ± 1.0 (1.0–6.0)	2.3 ± 1.1 (1.0–5.8)	0.226
No. of decompression levels			
1	20	91	0.080
2	11	86	0.374
3, 4, 5	3	36	0.230
Surgical methods			
BPL	18	111	0.928
LSPSL	10	81	0.333
MEL	6	21	0.177
Mean op time (range), minutes	153.0 ± 76.5 (55–381)	133.4 ± 51.1 (50–333)	0.266
Mean blood loss (range), g	87.9 ± 102.9 (0–360)	81.5 ± 98.0 (0–530)	0.945

**Table 3:** Characteristics of the patients who underwent reoperation. Op, operation; BPL, bilateral partial laminectomy; LSPSL, lumbar spinous process-splitting laminectomy; MEL, microendoscopic laminotomy. Mean values are presented as mean ± SD.

Characteristic	Reoperation	Non-reoperation	P-value
Mean age at op (range), years	69.9 ± 8.4 (54–79)	73.4 ± 9.3 (38–93)	0.186
Sex (male: female)	4:5	144:94	0.490
Duration of follow-up (range), years	3.0 ± 1.5 (1.25–6.0)	2.3 ± 1.1 (1.0-5.8)	0.121
No. of decompression levels			
1	4	107	0.976
2	5	92	0.308
3, 4, 5	0	39	0.186
Surgical methods			
BPL	4	125	0.634
LSPSL	3	88	0.824
MEL	2	25	0.269
Mean op time (range), minutes	134.1 ± 63.4 (57–238)	135.1 ± 55.3 (50–381)	0.789
Mean blood loss (range), g	90.0 ± 125.4 (0–350)	82.1 ± 97.6 (0–530)	0.703

Characteristics of the patients who progressed slippage

Postoperative progression of slippage at the same spinal level was observed in 35 patients (14.2%). Both progression and nonprogression of slippage groups were comparable in terms of age at the time of surgery, duration of follow-up, number of decompression levels, operative time, blood loss volume, or reoperation. Conversely, a greater progression of slippage existed in females than in males in terms of sex (P = 0.039). Additionally, LSPSL showed significantly less progression of slippage, while BPL showed significantly more progression of slippage (P = 0.026 and P = 0.037, respectively). The progression of DLSD significantly increased in patients who progressed slippage (P = 0.003; Table 4).

**Table 4:** Characteristics of the patients who progressed slippage. Op, operation; BPL, bilateral partial laminectomy; LSPSL, lumbar spinous process-splitting laminectomy; MEL, microendoscopic laminotomy. Mean values are presented as mean ± SD.

Mean age at op (range), years	72.6 ± 9.3 (42–88)	73.4 ± 9.3 (38–93)	0.572
Sex (male: female)	15:20	133:79	0.039
Duration of follow-up (range), years	2.5 ± 1.1 (1.0–6.0)	2.3 ± 1.1 (1.0–5.8)	0.074
No. of decompression levels			
1	15	96	0.789
2	16	81	0.139
3, 4	4	35	0.445
Surgical methods			
BPL	24	105	0.037
LSPSL	7	84	0.026
MEL	4	23	0.919
Mean op time (range), minutes	141.9 ± 57.3 (57–334)	136.7 ± 56.0 (46–381)	0.530
Mean blood loss (range), g	110.4 ± 105.7 (0–400)	77.8 ± 96.7 (0–530)	0.053
Progression of lumbar spine degeneration	11	23	0.003
Reoperation	3	6	0.120

DLSD with slippage progression was observed in one out of six patients (16.7%) with disc herniation, four out of seven patients (57.1%) with canal stenosis, five out of 13 patients (38.5%) with foraminal stenosis, and one out of eight patients (12.5%) with facet joint cyst. Meanwhile, 24 (11.3%) patients showed slippage progression without progression of DLSD. Patients with the progression of slippage had a higher likelihood of developing canal stenosis and foraminal stenosis (P < 0.001 and P = 0.010, respectively). However, no cases of canal stenosis leading to reoperation were observed. Conversely, patients who did not exhibit progression of DLSD demonstrated significantly less progression of slippage (P = 0.001; Table 5).

**Table 5:** The progression of DLSD with and without progressed slippage. DLSD, degenerative lumbar spinal disease.

DLSD (reoperation)	Progression of slippage	No progression of slippage	P-value
Disc herniation	1 (0)	5 (0)	0.859
Canal stenosis	4 (0)	3 (0)	<0.001
Foraminal stenosis	5 (3)	8 (5)	0.010
Facet joint cyst	1 (0)	7 (1)	0.890
None	24	189	0.001

Differences in surgical methods and progression of DLSD

Considering that MEL was performed at a single spinal level in all but one case, we accordingly studied the effects of different surgical methods on the postoperative progression of lumbar spine degeneration at a single level. A total of 111 patients underwent single-level decompression, comprising 62 BPL cases, 23 LSPSL cases, and 26 MEL cases. The progression of DLSD was found in 20 patients who underwent single-level decompression: 11 (17.7%) in BPL (disc herniation = 5; canal stenosis = 3; foraminal stenosis = 1; facet joint cyst = 2); three (13.0%) in LSPSL (disc herniation = 0; canal stenosis = 0; foraminal stenosis = 1; facet joint cyst = 2); and six (23.1%) in MEL (disc herniation = 0; canal stenosis = 3; foraminal stenosis = 2; facet joint cyst = 1). No significant differences were found in the progression of DLSD between the three single-level decompression surgical methods. Among the patients who had undergone single-level decompression, four required reoperation: one (1.6%) in BPL (disc herniation = 0; canal stenosis = 0; foraminal stenosis = 1; facet joint cyst = 0); one (4.3%) in LSPSL (disc herniation = 0; canal stenosis = 0; foraminal stenosis = 1; facet joint cyst = 0); and two (7.7%) in MEL (disc herniation = 0; canal stenosis = 0; foraminal stenosis = 1; facet joint cyst = 1). Similar to the results of the progression of DLSD, no significant differences were found between the three surgical procedures concerning reoperation (Table 6).

**Table 6:** The progression of DLSD after different surgical methods. DLSD, degenerative lumbar spinal disease; BPL, bilateral partial laminectomy; LSPSL, lumbar spinous process-splitting laminectomy; MEL, microendoscopic laminotomy.

Surgical methods	Progression of DLSD	Non-progression of DLSD	P-value	Reoperation	Non-reoperation	P-value
BPL	11	51	0.932	1	61	0.206
LSPSL	3	20	0.486	1	22	0.830
MEL	6	20	0.443	2	24	0.201

Timing of symptom onset and reoperation rates associated with the progression of DLSD

**Figure 3:**
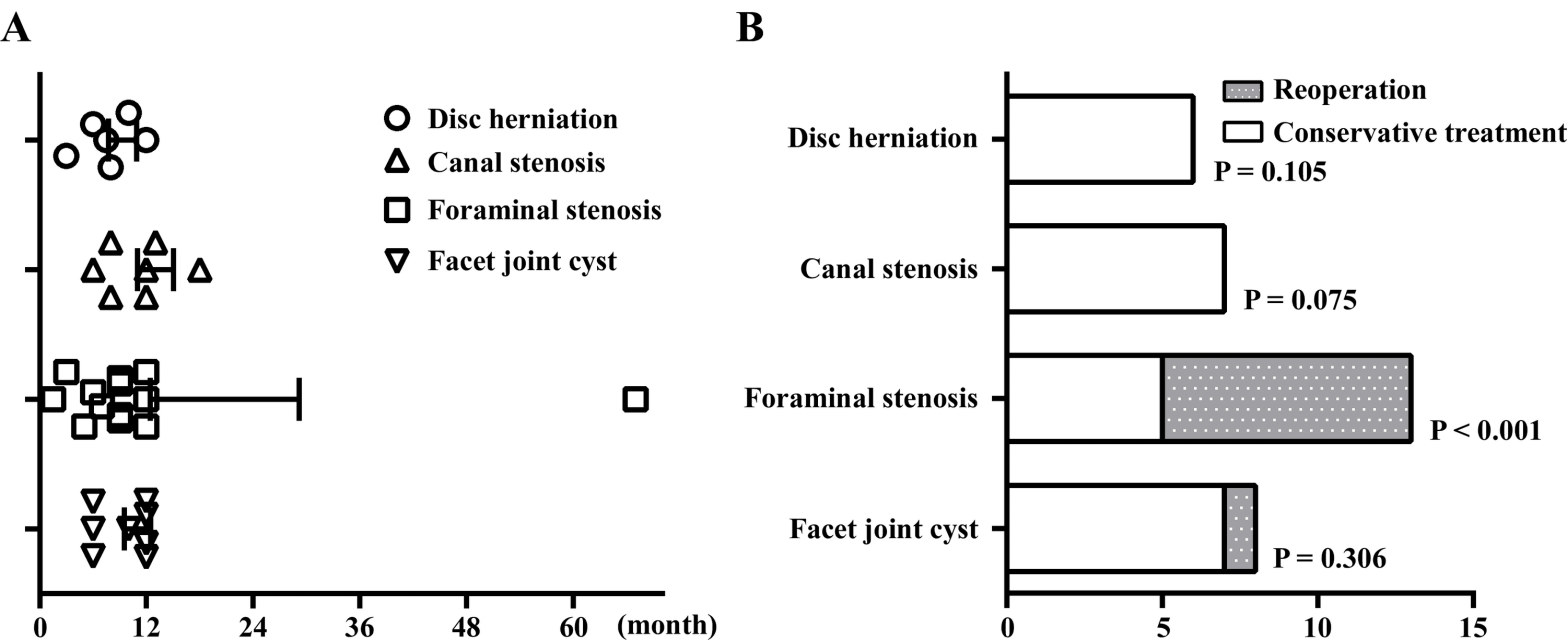
Timing of symptom onset and reoperation rates associated with the progression of degenerative lumbar spinal disease.

The onset of symptoms associated with the progression of lumbar spine degeneration was an average of 10.6 ± 10.5 months postoperatively (disc herniation = 7.8 ± 3.1 months; canal stenosis = 11.0 ± 4.0 months; foraminal stenosis = 12.4 ± 16.7 months; facet joint cyst = 9.5 ± 3.0 months). Among the 34 patients, 31 (91.2%) developed symptoms within one year (Figure 3A), out of whom nine patients underwent reoperation: eight cases (61.5%) of foraminal stenosis, and one case (12.5%) of facet joint cyst. The mean period from first surgery to reoperation was 18.8 ± 19.3 months in reoperation cases (the onset was an average of 14.3 ± 20.1 months postoperatively). Four out of the nine patients (44.4%) underwent reoperation within a year. The reoperation rates were significantly higher in patients with foraminal stenosis (P < 0.001; Figure 3).

## Discussion

Posterior lumbar spine decompression is a common procedure for treating spinal diseases, including LCS and spondylolisthesis. We observed a small number of cases in which the progression of DLSD occurred postoperatively, necessitating reoperation. Although many reports of reoperation due to postoperative instability and lumbar disc herniation exist [20-22], a few studies have examined cases that did not require reoperation [6,15]. In the present study, we retrospectively evaluated the progression of lumbar spinal degeneration after laminectomy for LCS, the risk factors associated with it, and the need for reoperation.

In this study, postoperative progression of DLSD was observed in 13.8% (34/247) of the patients, with 3.6% (9/247) requiring reoperation (Tables 1, 2). Over 90% of the instances of symptom onset (31/34) occurred within a one-year timeframe (Figure 3A). More than 40% of reoperations (4/9) were also performed within a year. Previous reports have indicated that reoperation rates after lumbar posterior decompression surgery ranged from 6.5% to 7%, making our results relatively modest [7,10,17,20,21,23,24]. The reoperation rates within one year ranged from 2% to 6% [10,23,24], similar to those in the present study (1.6%; 4/247). Therefore, particular attention should be paid to the progression of lumbar spinal degeneration in the first year after laminectomy. In this study, patients with foraminal stenosis had a particularly high reoperation rate (61.5%; Figure 3B). Therefore, if patients develop foraminal stenosis during the postoperative course, the possibility of reoperation should be considered. Furthermore, no differences were observed in the progression of DLSD or reoperation rate regardless of the number of decompression levels (Tables 2, 3). This is consistent with previous studies, including that by Javalkar et al. [20], who found no significant difference in the reoperation rates between patients with single-level and multiple-level stenosis, suggesting that the number of decompression levels may have little influence on degenerative disease progression.

Regarding the spinal structures that contributed to spinal stability, the proportions were as follows: facet capsule = 39%; intervertebral disc and annulus = 29%; supraspinous and interspinous ligaments = 19%; ligamentum flavum = 13% [25]. To minimize postoperative instability and achieve favorable outcomes, various surgical techniques have been proposed that aim to preserve the posterior spinal elements, including the lamina, spinous processes, interspinous ligaments, and facet joints [9,10,12,16]. In a previous report, radiographic analysis showed that patients who underwent LSPSL demonstrated lower slippage progression rates than those who underwent conventional laminectomy [10]. Additionally, Ramhmdani et al. reported that 1.6-32% of patients developed new or worsening spondylolisthesis after open lumbar decompression [26]. Similarly, the progression of postoperative slippage in the present study was observed in 14.2% of patients; however, it was reduced by LSPSL. Conversely, BPL showed significantly more progression of slippage (Table 4). The risk of slippage progression has been reported to increase in multilevel decompression because it involves the removal of more structural components [27]. However, in this study, no significant differences were observed in slip progression as the number of decompression levels increased (Table 4). The progression of slippage significantly exacerbated the organic stenosis of the spinal canal and intervertebral foramen; therefore, patients who did not exhibit slippage progression were less likely to consequently progress DLSD (Table 5). LSPSL inhibits the progression of slippage, potentially reducing the development of spinal canal and foraminal stenosis.

This study had some limitations. First, the number of cases varied across the different surgical methods because the choice of the procedure depended on the surgeon. Future studies should examine a uniformly distributed sample size. Second, regarding the follow-up period, previous reports indicated that DLSA progresses over time, which correlated with an increase in reoperation rates [21,23]. However, the present study included numerous cases with a follow-up period of only one year. Therefore future studies should consider longer follow-up periods to gain a clearer picture of the long-term effectiveness of these procedures and their complications.

## Conclusions

We investigated the progression of DLSD in patients who underwent decompression surgery for LCS. LSPSL reduces the incidence of canal stenosis and foraminal stenosis by suppressing postoperative spinal instability. Furthermore, the onset of symptomatic DLSD was more common within one year after surgery, and foraminal stenosis required reoperation in a higher proportion of cases. These findings could serve as guidance for spine surgeons in selecting the appropriate surgical methods for LCS, managing the symptoms of postoperative progression of DLSD, and evaluating the need for reoperation.
